# Velocity of centre of pressure as a predictor of walking speed

**DOI:** 10.1186/1757-1146-5-S1-O28

**Published:** 2012-04-10

**Authors:** Noël LW Keijsers, Niki M Stolwijk, Jan-Willem K Louwerens, Jaak Duysens

**Affiliations:** 1 Department of RD&E, Sint Maartenskliniek, Nijmegen, the Netherlands; 2 Orthopaedics, Sint Maartenskliniek, Nijmegen, the Netherlands; 3 Research Center for Movement Control and Neuroplasticity, Department of Biomedical Kinesiology, Katholieke Universiteit Leuven, Leuven, Belgium

## Background

Walking speed is one of the best measures of overall walking ability. In plantar pressure measurements, walking speed is usually assessed using a stopwatch, photocells, or camera systems. As a simple alternative, contact time can be used but contact time and walking speed are only moderately correlated. Because walking speeds alters foot loading, the centre of pressure might be of more value to indicate walking speed. Therefore, the purpose of this study is to assess walking speed using the velocity of the centre of pressure (VCOP).

## Materials and methods

Thirty-three subjects (range 19-77 years) walked over a Foot Scan pressure plate (Rsscan Int) at three speed conditions; slow, preferred, and fast. Contact time and mean VCOP during stance were calculated for each subject and condition. Walking speed was measured by a Vicon motion analysis system using a marker on the heel. Linear regression analysis was used to indicate the relation between walking speed and the independent variables: contact time, mean VCOP during stance, and both combined for all walking conditions separately and together. Finally, stance phase was divided in 5 equal time frames; the mean VCOP was calculated for each frame and used as 5 independent variables (5 VCOP)

## Results

When all walking speeds were combined, walking speed was highly correlated with mean VCOP and contact time (see Table 1). However, correlation coefficients values were much lower for the preferred, low, and fast walking speed separately. Mean VCOP showed larger correlation coefficients than contact time. Adding contact time, the correlation coefficient increased minimally. Walking speed was best predicted when the 5 VCOP values were used.

**Table 1:** Correlation coefficient values (r)

Walking speed	Mean VCOP	Contact time	Both	5 VCOP
Slow	0.80	-0.78	0.82	0.84
Preferred	0.90	-0.69	0.90	0.91
Fast	0.60	-0.45	0.61	0.76
All walking speeds	0.94	-0.88	0.94	0.96

## Conclusions

Mean VCOP is a better predictor for walking speed than contact time. Using more detailed information of the VCOP, the prediction of walking speed can be further increased.

